# Remote Interdisciplinary Care for Atopic Dermatitis Using a Digital Care Concept (ADCompanion): Protocol for a Multicenter Randomized Trial

**DOI:** 10.2196/88025

**Published:** 2026-08-20

**Authors:** Stephanie Dramburg, Camilo José Hernandez Toro, Josephine Telschow, Susanne Abraham, Meslina Almaci, Anna Behringer, Simone Christel, Angela Cornelius, Janina Feustel, Sascha Fischer, Isabelle Gloeyer, Nadia Günther, Katharina Hagemeister, Eckard Hamelmann, Leonard Harries, Annice Heratizadeh, Nora Knappe, Sonja Lämmel, Eva Moennig, Irena Neustädter, Christina Pham, Laura Rechtien, Thomas Reinhold, Christina Schnopp, Christina Schorlemer, Lukas Sollfrank, Sophie Spies, Kristina Stamos, Michael Sticherling, Piera Tierno, Marion Trentmann, Marie-Therese Unterweger, Christian Vogelberg, Hanna Winkler, Margitta Worm, Mehrak Yoosefi, Ulrike Grittner

**Affiliations:** 1 Department of Pediatric Respiratory Care, Immunology and Intensive Care Medicine, Charité – Universitätsmedizin Berlin, Augustenburger Platz 1, Berlin, 13353, Germany, 49 30450 559839; 2 Institute of Biometry and Clinical Epidemiology, Charité – Universitätsmedizin Berlin, Berlin, Germany; 3 Department of Dermatology, University Allergy Center, Medical Faculty Carl Gustav Carus, Technische Universität Dresden, Dresden, Germany; 4 Division of Allergy and Immunology, Department of Dermatology, Venerology and Allergy, Charité – Universitätsmedizin Berlin, Berlin, Germany; 5 Department of Dermatology and Allergology Biederstein, Klinikum Rechts der Isar, Technical University of Munich, Munich, Germany; 6 Department of Dermatology, Friedrich-Alexander-Universität Erlangen-Nürnberg, Erlangen, Germany; 7 Department of Dermatology and Allergy, Medizinische Hochschule Hannover, Hannover, Germany; 8 Department of Pediatrics, Children's Center Bethel, University Hospital OWL, Bielefeld University, Bielefeld, Germany; 9 Department of Pediatrics, Medical Faculty Carl Gustav Carus, University Allergy Center, Technische Universität Dresden, Dresden, Germany; 10 Deutscher Allergie- und Asthmabund e.V. (German Allergy and Asthma Association e.V.), Mönchengladbach, Germany; 11 Department of Paediatrics, Cnopf´sche Kinderklinik, Nuremberg, Germany; 12 Institute for Social Medicine, Epidemiology and Health Economics, Charité – Universitätsmedizin Berlin, Berlin, Germany

**Keywords:** atopic dermatitis, telemedicine, video consultation, patient education, nurse-led care, psychosocial support, nutritional counseling, digital health intervention, interprofessional care, chronic disease management, eHealth, counseling

## Abstract

**Background:**

Atopic dermatitis (AD) is one of the most common chronic inflammatory skin diseases, associated with itching, sleep disturbance, and impaired quality of life. Structured patient education can improve disease outcomes, but in-person programs are often unavailable in rural or underserved areas. Digital interventions may help overcome these barriers; however, their efficacy compared with routine care has not been evaluated.

**Objective:**

This study aims to assess whether a digitally delivered, interdisciplinary care concept (ADCompanion) is noninferior to routine, real-world care, as available in well-served areas, in reducing disease severity among patients with AD. Secondary objectives include effects on quality of life, pruritus, psychosocial burden, family well-being, and health care costs.

**Methods:**

This is a prospective, multicenter, 2-arm, 1:1 randomized noninferiority trial. Participants are stratified by age and disease severity. The intervention group receives standard therapy plus access to a digital platform with training modules and optional video consultations on skin care, nutrition, and psychosocial support. The control group receives routine care, optionally including face-to-face group training where available, and a basic app version for symptom tracking. The primary endpoint is physician-assessed disease severity (Scoring Atopic Dermatitis Index [SCORAD]) at 6 months; secondary endpoints include patient-reported outcomes, itch, quality of life, and disease-related costs.

**Results:**

Recruitment of 603 participants across 7 centers in Germany was completed. Data collection was finalized in June 2025. At the time of protocol submission (February 2026), data cleaning had been completed, and the statistical analysis plan was finalized; final results are expected to be published in 2026.

**Conclusions:**

This protocol describes a randomized trial designed to evaluate whether digitally delivered interdisciplinary care is noninferior to routine care for patients with AD. The findings of this study will inform the role of digital patient education as a complementary component within established care structures.

## Introduction

Atopic eczema (atopic dermatitis, AD) is a chronic skin disease with a prevalence of up to 20% in children, depending on the age group [1], and up to 7% of adults [2]. The disease is characterized by chronic inflammation leading to often tormenting itch and chronic skin rashes with potential superinfection. AD often severely impacts the quality of life and sleep of patients and their social environment, particularly when children are affected [3]. Further, an association between early AD and the development of psychiatric disorders such as attention-deficit hyperactivity disorder or autistic spectrum disorders has been described in several studies [4,5]. Among adult patients, an increased prevalence of depression and an average loss of work productivity of 6% have been recently reported in a large registry trial [6]. Despite the availability of effective systemic therapies for moderate-to-severe AD, appropriate topical treatment and daily self-management of flares remain essential components of long-term disease control. The need among patients and caregivers for comprehensive information about the illness and possible forms of treatment and management is often not adequately met during physician consultations, which leads to uncertainty when dealing with important issues such as self-management or stigmatization. As chronic diseases with recurrent exacerbations pose substantial challenges for both patients and health care professionals, specific patient training programs are a key element of several guidelines on AD [7-10]. In Germany, a validated curriculum for group training sessions has been developed, providing multidisciplinary care for individuals and families affected by AD. Its positive impact on the course of disease and quality of life was observed in a pediatric cohort [11] and an adult cohort [12]. A recent scoping review on patient training formats for AD identified 16 studies on different formats such as group-based educational programs, individual consultations with specialized health care providers, pharmacists’ counseling, educational brochures, and online resources [13]. Despite methodological heterogeneity, all concepts resulted in better outcomes compared to no training. Most proposed training programs are designed as in-person group-based formats, taught by an interdisciplinary group of specifically trained health care professionals [11-18]. Although showing positive results, this comprehensive approach requires the availability of qualified staff on site, which can be a limiting factor, particularly in underserved regions. Although during and after the SARS-CoV-2 pandemic, remote care delivery increased exponentially and AD training programs, among others, were delivered online [19], comparative evidence between different care formats remains scarce.

Despite guideline recommendations and established training concepts, long-term disease control in AD frequently remains suboptimal. This is partly driven by challenges in day-to-day self-management, including treatment adherence, uncertainty about trigger management and skincare routines, corticosteroid concerns, and limited opportunities for reinforced counseling in routine visits [20,21]. Consequently, contemporary national and international guidelines emphasize structured patient and caregiver education as a core component of AD management [7,8,10]; however, access to multiprofessional educational programs remains heterogeneous and often limited by local availability, staffing resources, and organizational barriers [22,23].

Digital health interventions may help address these access limitations by delivering evidence-based education and counseling independent of geographical location [24]. Over the past few years, online behavioral and self-management interventions for eczema have shown modest improvements in patient-reported outcomes; however, many digital interventions primarily rely on self-assessed disease severity, while comparative data using clinician-assessed outcomes remain limited [22,23,25]. Beyond patient-level outcomes, unmet needs also affect caregivers and health care professionals involved in AD management, particularly in the context of limited consultation time and fragmented care structures. Families frequently report difficulties in coordinating care, navigating treatment recommendations, and managing disease-related psychosocial stress in everyday life. From a health care provider's perspective, limited consultation time, high demand, and fragmented care structures constrain the ability to deliver repeated, individualized education and follow-up support. Digital care concepts that combine standardized educational content with flexible, needs-based counseling may therefore help reduce structural barriers while supporting continuity of care across sectors and professional groups [10,23,26].

Building on validated multiprofessional educational curricula [11,12], and combining asynchronous education with symptom and trigger tracking as well as optional synchronous video consultations, *ADCompanion* was developed as a comprehensive interdisciplinary digital care concept for patients with AD across age groups. Against this background, this study evaluates whether this digitally delivered intervention achieves noninferior clinical outcomes compared with routine care in well-served regions.

This paper describes the study protocol of a prospective, multicenter, 1:1 randomized 2-arm intervention study with a noninferiority design to test whether the digitally delivered concept of care is equally effective in improving eczema severity (primary objective), pruritus, quality of life, family well-being, and health care–related costs (secondary objectives).

This paper reports the study protocol of the ADCompanion trial and is published after completion of recruitment and data analysis (Registered Report stage DERR1) in accordance with JMIR Protocols guidelines.

## Methods

### Study Aims

ADCompanion aims to evaluate a remote care concept for AD to positively influence the course of disease through (1) digitization of selected and validated patient training content, (2) standardized symptom and trigger recording, as well as optional individual data-supported video consultations in the areas of (3) nutrition, (4) skin care, and (5) psychosocial support. Via this digitally delivered care concept, the authors also aim to improve the quality of life and health literacy of patients/caregivers to ensure long-term participation in the social and professional/school context.

### Study Design

ADCompanion is a prospective, multicenter, 1:1 randomized 2-arm intervention study with a noninferiority design. Patient enrollment is stratified in 3 age groups: infants, toddlers, and preschool children (age 3 months to 6 years); school children and adolescents (age 7‐17 years); and adults (age 18‐65 years). The need for this stratification results from known differences in care between age groups and a high spontaneous remission rate in preschool age.

**Figure 1.**
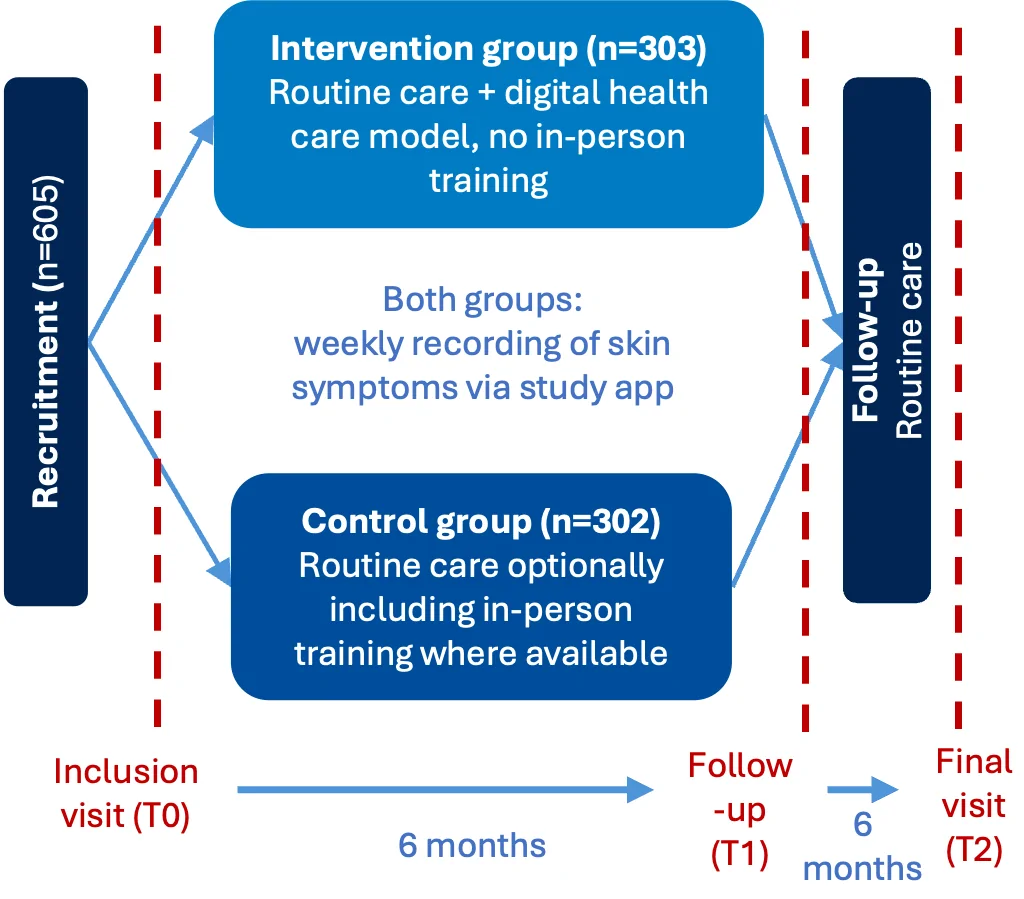
Overview of the study timeline and visits.

The study groups differ as follows: The control group receives standard therapy (optionally including access to face-to-face training in small groups where possible, in line with routine care) and access to a limited version of the study app containing standardized symptom and trigger recording only. Routine care in this trial reflects routine real-world care as delivered across participating centers and does not mandate participation in structured face-to-face education programs. The intervention group receives standard medical therapy without access to face-to-face group training, but with full use of the app, including online training content and optional individual counseling via video in the areas of care, nutrition, and psychosocial support. Participants can freely decide which counseling sessions they want to book. Per specialist area, a maximum of 3 sessions can be booked during the first monitoring period (6 months). For an overview on the study visits and timeline, please see Figure 1. Due to the nature of the intervention, blinding of participants or investigators was not feasible and therefore not applied.

Patients were recruited in the outpatient departments of 7 German hospitals—Berlin (pediatric and dermatology departments), Bielefeld (pediatric department), Dresden (pediatric and dermatology departments), Erlangen (dermatology department), Hannover (dermatology department), Munich (dermatology department), and Nürnberg (pediatric department). The study has been approved by all local ethics committees and is registered at the German Clinical Trials Register (ID: DRKS00030902).

This study received public funding from the Innovation Fund of the Federal Joint Committee of Germany, which serves as a central policy instrument to advance health care delivery in Germany.

### Participants and Recruitment

Patients with a physician-confirmed diagnosis of AD were recruited consecutively between May 2023 and May 2024 from the outpatient departments of 7 university and community hospitals across Germany (Berlin, Bielefeld, Dresden, Erlangen, Hannover, Munich, and Nuremberg). Potential participants were identified during routine visits and informed about the study by trained study personnel. Additionally, flyers and posters that informed about the study were placed in doctors’ waiting rooms, hospital hallways, and preschools. The statutory health insurance and project partner “Techniker Krankenkasse” further sent out 73,000 informative leaflets to clients with a diagnosis of atopic eczema. After providing written informed consent (or parental consent for minors), participants were screened for eligibility criteria (Textbox 1). Ongoing systemic therapies were no exclusion criterion, as long as a Scoring Atopic Dermatitis Index (SCORAD) of ≥20 points was reached. To promote retention, a small compensation fee of €30 (US $34.72) for participants of the intervention and €60 (US $68.73) for patients in the control group was given after completion of the final study visit.

Textbox 1. **Criteria for inclusion and noninclusion.**

**Inclusion criteria**

- Age 3 months to 17 years (children/adolescents) or 18-65 years (adults)
- A medically confirmed diagnosis of atopic dermatitis (AD) that has existed for at least 3 months
- Moderate to severe AD (Scoring Atopic Dermatitis Index [SCORAD] of ≥20 points)
- Stable treatment regimen for the last 3 months (eg, no new use of systemic therapies planned)
- Possession of an internet-enabled smartphone (for children under 14 years of age, possession of the smartphone by the parents is decisive)
- Confident command of the German language (for young children, the parents’ command of the German language is decisive)
- Enrollment in German statutory health insurance

**Exclusion criteria**

- Participation in a rehabilitation or training program for AD in the last 2 years
- Participation in individual counseling pertaining to AD in the areas of care, nutrition, and/or psychosocial support in the last 12 months
- Planned, significant change in treatment regimen (eg, first-time use of biologics) in the next 6 months
- Other serious illnesses that may have an impact on the skin condition (eg, autoimmune diseases)

### Randomization

Randomization was performed according to the following strata in a 1:1 ratio in the intervention and control groups: (1) study centers, (2) age (children aged 3 months to 6 years, children aged 7‐17 years, and adults aged 18‐65 years), and (3) disease severity (patients with a SCORAD of 20‐50 points were categorized as mildly/moderately and patients with a SCORAD of >50 points as severely affected).

Due to the high number of strata, unbalanced results may occur in a simple randomization with regard to the stratification variables between the intervention group and the control group. To ensure balanced treatment assignment across multiple prognostic factors, participants were assigned to either the intervention or control group (1:1 ratio) using a deterministic variance minimization algorithm [27]. The stratification factors included study center, age (3 months to 6 years, 7‐17 years, and 18‐65 years), and baseline SCORAD (cutoff of 50 points). While the algorithm did not use a random element (eg, a biased coin), allocation concealment was strictly maintained as the automated assignment was generated by a web platform only after the successful completion of the baseline assessment and recruitment. Given the complexity of the balancing across numerous strata and the centralized, software-based nature of the allocation, the treatment path was not accessible to the investigating clinicians, thereby minimizing the risk of selection bias. Randomization took place after informed consent, after download of the study app, and after the baseline survey via computer software, with no influence of investigators. Due to the app-based onboarding process and immediate assignment within the digital system, full blinding was not feasible; however, treatment allocation could not be influenced by study personnel.

### Intervention

ADCompanion strengthens the holistic care of patients with AD in underserved regions through location-independent access to the following interdisciplinary care components.

#### Online Access to Qualified Training Content and Standardized Recording of Symptoms and Trigger Factors

**Figure 2.**
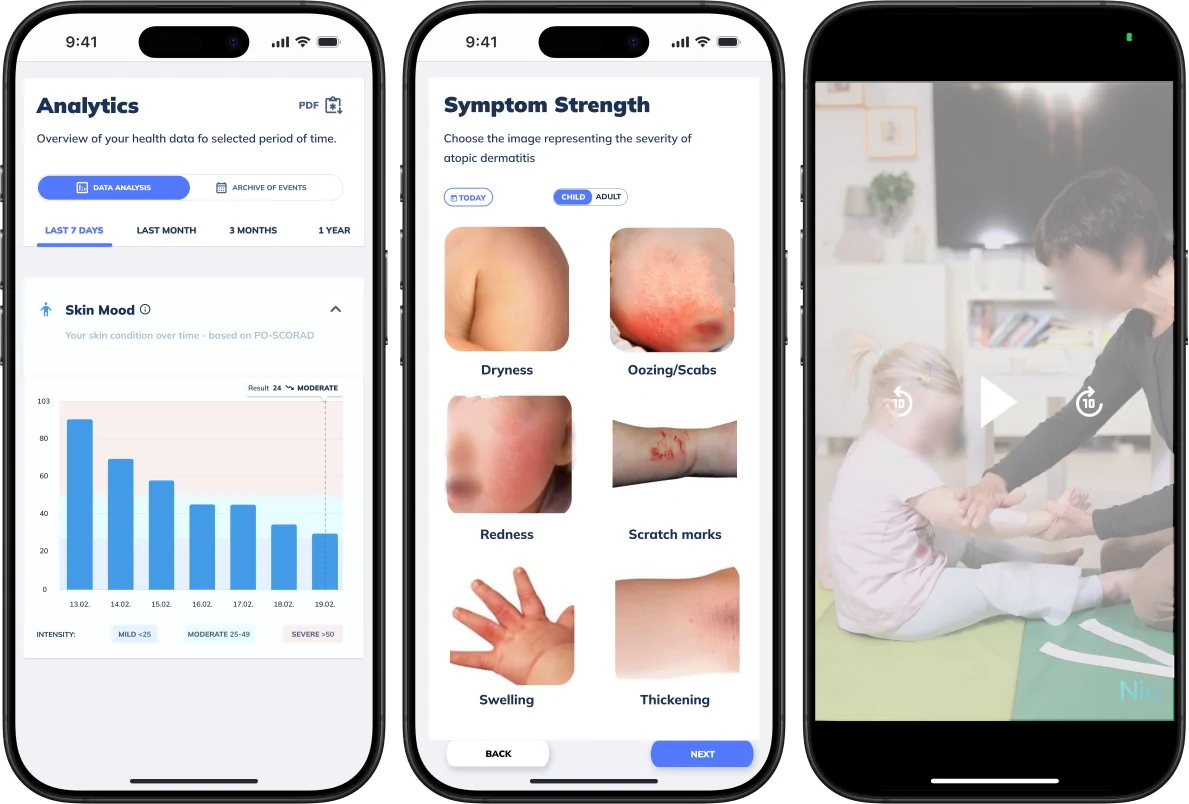
Screenshots of the study app including video and text material from the AGNES training curriculum. AGNES: Arbeitsgemeinschaft Neurodermitisschulung e.V. (German Working Group on Atopic Dermatitis Training for Children).

This part of the intervention was delivered via a specifically modified version of the publicly available app “NIA” (Nia Health GmbH). In the project-specific app version (not publicly available), ADCompanion offers content from the corresponding patient educational curricula of the “Arbeitsgemeinschaft Neurodermitisschulung” (AGNES, German Working Group on Atopic Dermatitis Training for Children) [11] and “Arbeitsgemeinschaft Neurodermitisschulung für Erwachsene” (ARNE, German Working Group on Atopic Dermatitis Training for Adults) [12], which have been edited in cooperation with teams of experts to adapt them for digital and remote communication. Prepared texts, expert interviews, and video tutorials convey specialist knowledge in easy-to-understand language and clarify common questions from those affected. The project app also enables the standardized recording of “skin mood” (dryness, oozing/crusting, redness, scratching, swelling, and skin thickening) via the validated Patient-Oriented Scoring Atopic Dermatitis Index (PO-SCORAD) [28]. In addition, possible trigger factors can be recorded and skin conditions documented using photos. Individual reports can be downloaded in a .pdf format and shared with the attending health care professionals. For screenshots of the study app, please see Figure 2. Use of the digital intervention was intentionally not restricted or prescribed in order to assess real-world utilization. Participants in the intervention group received full access to all app components and were free to decide how often and which elements to use. No minimum number of logins, completed modules, or counseling sessions was defined to constitute intervention adherence.

#### Needs-Based, Qualified Nutritional Advice via a Digital Consultation Platform

As AD is often associated with sensitization to various foods, especially in childhood, uncertainties regarding the correct diet are common and malnutrition can occur. To counteract this risk and support the adequate implementation of an elimination diet, ADCompanion offers the option of qualified nutritional therapy advice via video.

#### Individual Video Care Advice via Digital Consultation Platform

The needs-adapted skin care advice by specifically qualified nurses (AGNES trainer certification) is provided in accordance with the validated care advice initiative of AGNES e.V. [29].

#### Individual, Psychosocial Counseling on Dealing With the Disease and Possible Coping Strategies

AD patients of all age groups and their caregivers/families often face major challenges, particularly due to the pronounced itching, but also cosmetic aspects and restrictions in everyday life, work, and sleep. ADCompanion offers the possibility of needs-adapted support by means of video counseling by specifically qualified psychologists (AGNES trainer certification).

### Primary Hypothesis

Patients in the intervention group have a comparable or lower severity of AD, as measured by doctor-assessed SCORAD [30], than patients in the control group 6 months after the start of intervention (noninferiority).

### Primary Outcome

The primary outcome of the study is the change in physician-assessed SCORAD index [31] 6 months after baseline. SCORAD is a validated composite instrument combining the extent of affected skin areas, the intensity of 6 clinical signs, and patient-reported symptoms (pruritus and sleep loss), resulting in a total score ranging from 0 to 103, with higher scores indicating more severe disease.

### Secondary Outcomes

Secondary outcomes capture patient- and caregiver-reported disease severity, symptoms, quality of life, psychosocial burden, family impact, and digital health literacy. Subjective disease severity is assessed using the PO-SCORAD [28], a self-assessment version of SCORAD aligned with the clinician-rated instrument. In addition, clinician-rated disease severity is measured using the Eczema Area and Severity Index (EASI) [31], which evaluates the extent and intensity of eczema lesions across 4 body regions and yields a total score ranging from 0 to 72.

Dermatology-specific symptom burden and quality of life are assessed using the German version of the SKINDEX-29 (29-Item Dermatology-Specific Quality of Life Questionnaire) [32], which includes symptom, emotional, and functional domains scored on a 0‐100 scale, with higher scores reflecting greater impairment. Itch-related cognitions and coping strategies are assessed using age-appropriate validated German instruments, namely JUCKKI/JUCKJU (Itch Questionnaire for Children/Itch Questionnaire for Adolescents) for itch-related cognitions and COPEKI/COPEJU (Coping With Disease Questionnaire for Children/Coping With Disease Questionnaire for Adolescents) for coping with itch and disease-related stress [33].

For infants and toddlers, health-related quality of life is assessed using the Infants’ Dermatitis Quality of Life Index (IDQoL) [34], a parent-reported questionnaire with total scores ranging from 0 to 30. The impact and burden of AD on family life is measured using the Familien-Belastungs-Fragebogen (FaBel) [35].

Digital health literacy is assessed using the eHealth Literacy Scale (eHEALS) [36], an 8-item self-report questionnaire with total scores ranging from 8 to 40, where higher scores indicate higher perceived competence in accessing and using digital health information.

Further secondary outcomes are the number of responders (SCORAD improvement of more than ≥8.7 points at 6 and 12 months compared to baseline [37]) and the association between the use of individual care components by patients and/or caregivers and the individual level of competence in dealing with digital health services/information (eHEALS questionnaire on digital health literacy, measured at baseline [27]).

All outcome measures, including assessment timepoints and target populations, are summarized in Table 1. Clinician-rated outcomes are assessed during study visits by trained study personnel, while patient- and caregiver-reported outcomes are collected electronically.

**Table 1.** Overview of tools and timepoints of data collection.

Variable	Timepoint of recording			
	Baseline (T0)	Weekly recording via study app	6 months after baseline (T1)	12 months after baseline (T2)
Primary outcome				
SCORAD^a^	✓		✓	
Secondary outcome				
SCORAD	✓			✓
EASI^b^	✓		✓	✓
PO-SCORAD^c^	✓	✓	✓	✓
SKINDEX-29^d^	✓		✓	✓
IDQOL^e^	✓		✓	✓
Impact on Family Scale	✓		✓	✓
JUCKKI^f^/JUCKJU^g^	✓		✓	✓
COPEKI^h^/COPEJU^i^	✓		✓	✓
eHEALS^j^	✓			
Medication diary	✓	✓	✓	✓
Questionnaire on the use of medical services	✓		✓	✓

a SCORAD: Scoring Atopic Dermatitis Index.

b EASI: Eczema Area and Severity Index.

c PO-SCORAD: Patient-Oriented Scoring Atopic Dermatitis Index.

d SKINDEX-29: 29-Item Dermatology-Specific Quality of Life Questionnaire.

e IDQOL: Infant’s Dermatology Quality of Life Index.

f JUCKKI: Itch Questionnaire for Children.

g JUCKJU: Itch Questionnaire for Adolescents.

h COPEKI: Coping With Disease Questionnaire for Children.

i COPEJU: Coping With Disease Questionnaire for Adolescents.

j eHEALS: eHealth Literacy Scale.

### Statistical Analysis

#### Analysis of Primary Outcomes

The statistical analysis of the main hypothesis of noninferiority will be performed using analysis of covariance (ANCOVA). This analysis framework also enables the evaluation of potential superiority if supported by the data. The primary outcome is the disease severity measured via change in SCORAD 6 months after study inclusion. Covariates are SCORAD at baseline and patient age. Center heterogeneity is accounted for by random intercepts using linear mixed models. Noninferiority for the overall study population is considered proven if the 95% CI of the mean group differences between the control and intervention groups determined by ANCOVA (linear mixed model) is less than 4 points in favor of the standard therapy. Once noninferiority has been demonstrated in the overall group, noninferiority is demonstrated in the individual age strata. For this purpose, the regression model is extended by an interaction term for the age groups and the allocation (intervention vs control). Based on marginal estimators, the 95% CIs for the group differences (intervention vs control) are then determined for the respective age strata. Noninferiority in the individual age strata is shown following the principle of hierarchical testing [38] using a family-wise error rate of 0.025 (one-sided) and applying the Bonferroni and Holm method [39] for the adjustment for multiple testing if (1) in the stratum with the strongest effect, the 98.3% (family-wise error rate of 0.025/3) CI of the marginal estimator for the group difference in the SCORAD is less than 4 points in favor of the standard treatment; (2) in the stratum with the second strongest effect, the 97.5% (family-wise error rate of 0.025/2) CI of the marginal estimator for the group difference in the SCORAD is less than 4 points in favor of the standard treatment; and (3) in the stratum with the smallest effect, the 95% (family-wise error rate of 0.025) CI of the marginal estimator for the group difference in the SCORAD is less than 4 points in favor of the standard treatment.

#### Analysis of Secondary Hypotheses

Secondary hypotheses are tested using similar statistical models. The dependence of repeated measurements is considered using mixed models (random intercept models). All hypotheses are analyzed in the full analysis set after multiple imputation according to the intention-to-treat approach. The analyses are adjusted for the respective baseline values, age, and SCORAD at baseline. Center heterogeneity is taken into account using random intercepts. An exploratory analysis will assess the potential superiority of medical routine care plus ADCompanion versus medical routine care alone (without in-person training).

In addition to the intention-to-treat analysis, exploratory per-protocol analyses will be conducted. For these analyses, the per-protocol population is defined based on actual use of the digital intervention (eg, app logins, symptom tracking entries, or participation in video counseling), as recorded by the app. These analyses are exploratory in nature and intended to describe the association between intervention uptake and outcomes rather than to redefine intervention exposure.

#### Dealing With Missing Values

Missing values are initially recorded and reported descriptively. If, after exploratory analyses, the assumption of “missing at random” or “missing completely at random” is reasonable, data are imputed using multiple imputation methods. The reasons for the missing values are documented and reported as well as possible. Nonimputed datasets are used in sensitivity analyses (complete case analysis).

#### Subgroup Analyses

To evaluate possible differential treatment effects in the predefined subgroups, interaction terms between treatment arm and subgroup are first included in the regression models described above. Treatment effects in the subgroups are also reported based on the regression models with interactions using marginal effect estimates and 95% CIs. In addition, the effect estimate for the interaction is reported with 95% CIs and *P* values. No interim analyses were planned.

#### Sample Size Calculation

The case number estimate was based on a noninferiority design and the primary endpoint SCORAD 6 months after baseline. The noninferiority margin was set at 4 points, as differences in SCORAD of 4 points or less are not considered clinically relevant [19]. Using a *t* test for independent samples with a power of 85% and a 1-sided significance level of 0.025, noninferiority of ADCompanion compared to standard therapy can be demonstrated, assuming a common SD of 15 points (from Staab et al [11] and Heratizadeh et al [12], a common (pooled) SD of 11.2 was calculated). Considering possible stronger variances in our study design, the power calculation was based on a common SD of 15 points if 254 patients per group are included. This corresponds to a total number of cases of 508 patients. Based on an expected dropout rate of about 15%, 600 patients should be included in the study (corresponding to a dropout rate of 15.3%). This case number estimation was performed with nQuery Advisor 8 (nQuery 2017 Sample Size and Power Calculation, “Statsols,” Statistical Solutions Ltd).

We assume that the intervention effects may be different in the various age groups. Since the spontaneous remission rates are much higher in younger children in particular, lower effect sizes are to be expected compared to older children and adults. Therefore, proportionally more patients should be recruited in the age group under 7 years. Staab et al [11] determined different effect sizes (Cohen *d*) in different age groups: 3 months to 7 years: 0.23; 8‐12 years: 0.35; 13‐18 years: 0.76. Due to the expected different effect sizes, age group–specific recruitment was determined as (1) age 3 months to 6 years: n=260 (130 IG + 130 CG); (2) age 7‐17 years: n=140 (70 IG + 70 CG); and (3) 18‐65 years: n=200 (100 IG + 100 CG), where CG represents the control group and IG represents the intervention group.

### Data Management

The data collected within the project framework includes personal data and questionnaire results (including SCORAD) collected during the study visits, as well as patients’ diary entries on skin appearance and possible trigger factors, which are continuously recorded using the study app.

The identifying data are recorded exclusively on paper at the inclusion visit and kept under lock and key by the recruiting centers for 10 years. The identifying data are assigned to the study identification numbers on the participant declarations and in an enclosed paper list. From this point on, the data collected for the project will only be collected and stored in pseudonymized form. When using the app, an identifying email address must be stored for technical support. However, this information remains with the app manufacturer and is not shared with any third party.

The questionnaires are collected electronically during the study visits using an electronic clinical record form created with the software SecuTrial. The recorded data are stored directly on Charité servers, which guarantees a high level of data security and the creation of regular backup copies. No study associated with digital data storage takes place in the study centers.

The data collected using the monitoring app will be exported after the follow-up visits (at 6 and 12 months) and transmitted for statistical analysis in an encrypted form.

### Ethical Considerations

The trial was approved by the ethics committees of all participating centers in Germany (Charité – Universitätsmedizin Berlin, Medical Faculty TU Dresden, Technical University Munich, Friedrich Alexander University Erlangen, Hannover Medical School, University Bielefeld, and Cnopfsche Kinderklinik Nuremberg) (Multimedia Appendix 1). Written informed consent was obtained from all adult participants and from parents or legal guardians of minors; in addition, age-adapted assent was collected from children and adolescents aged 8 years and older. All data are pseudonymized and handled in accordance with the EU General Data Protection Regulation (GDPR) and national data protection laws. No harm was expected for participants receiving the intervention and members of the control group.

No protocol amendments were necessary during the course of the study. The study information was regularly updated in the trial registry (DRKS00030902; registration date: May 22, 2023; protocol version 2.0, March 6, 2023). The study follows the principles of the Declaration of Helsinki and good clinical practice.

Study results will be disseminated through peer-reviewed publications, conference presentations, and summaries for participants and stakeholders. Authorship eligibility follows ICMJE criteria.

## Results

**Figure 3.**
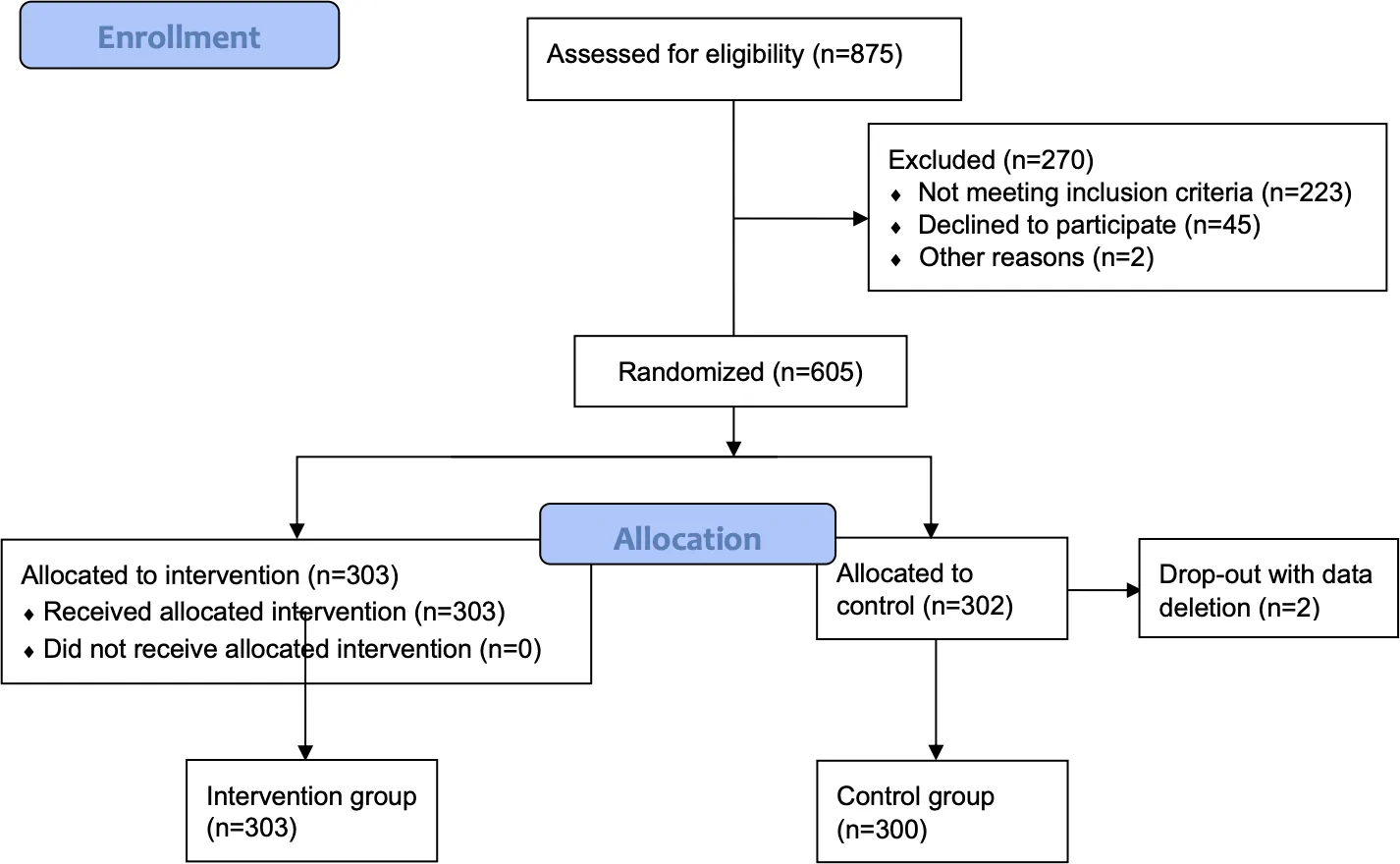
CONSORT (Consolidated Standards of Reporting Trials) flow diagram.

Recruitment was completed, and 605 participants were included in the study. Of these, 2 dropouts requested data deletion, leaving 603 participants for full-set data analysis: 252 infants and children aged 3 months to 6 years, 146 children and adolescents aged 7‐17 years, and 205 adults aged 18‐65 years (Table 2). Data collection was finalized in June 2025, and data analysis has been completed. Final study results will be reported in a separate results manuscript. The number of participants slightly exceeds the initially planned numbers due to parallel recruitment procedures in the study centers. Screening failures were not systematically recorded. However, several study centers reported a SCORAD below 20 points as a frequent reason for noninclusion. In most of these cases, the reported severity and/or individual experience was not reflected by corresponding skin lesions. For the CONSORT flowchart, please see Figure 3. At the time of protocol submission (November 2025), follow-up assessments were completed.

Baseline characteristics of the participants are shown in Table 2.

During the intervention period, of the 233 control group participants who completed their 6-month study visit (6 months after baseline), 24 (10.3%) participated in a specialized, multiprofessional, in-person group training for AD. Of those, 12 families with children, 8 adolescents (and/or their families), and 4 adults attended a face-to-face training session. Availability of face-to-face training differed across study centers and age groups.

**Table 2.** Baseline characteristics of the study population by treatment arm, selected variables.

	Overall (n=603)	Intervention (n=303)	Control (n=300)
Gender (female), n (%)	325 (53.9)	162 (53.5)	163 (54.3)
Age (years)			
Mean (SD)	15.96 (16.2)	16.17 (16.95)	15.75 (15.44)
Median (IQR)	9 (3-28)	9 (2-28)	9 (3‐26.25)
SCORAD^a^			
Mean (SD)	34.72 (11.64)	34.88 (11.33)	34.57 (11.97)
Median (IQR)	32 (26-41)	33 (26-41)	32 (25-40)
Level of therapy handling, n (%)			
Level 1: Topical emollient therapy	61 (10.1)	35 (11.6)	26 (8.7)
Level 2: Low-potency topical anti-inflammatories	350 (58)	178 (58.7)	172 (57.3)
Level 3: Higher-potency topical anti-inflammatories	177 (29.4)	84 (27.7)	93 (31)
Level 4: Systemic immunomodulatory therapy	14 (2.3)	5 (1.7)	9 (3)
N/A^b^	1 (0.2)	1 (0.3)	0 (0)
Family history of allergies, n (%)			
Overall	451 (74.8)	229 (75.6)	222 (74)
Allergic comorbidities	305 (50.6)	143 (47.2)	162 (54)
Allergic rhinitis	242 (40.1)	111 (36.6)	131 (43.7)
Asthma	74 (12.3)	35 (11.6)	39 (13)
Food allergy	145 (24)	71 (23.4)	74 (24.7)
Insect venom allergy	3 (0.5)	1 (0.3)	2 (0.7)
Medication allergy	16 (2.7)	8 (2.6)	8 (2.7)
Contact allergy	14 (2.3)	8 (2.6)	6 (2)
Other allergy	45 (7.5)	24 (7.9)	21 (7)
Previous allergy testing	387 (64.2)	184 (60.7)	203 (67.7)
Positive allergy test	311 (51.6)	144 (47.5)	167 (55.7)
Private, atopic dermatitis–related expenses in the past 6 months			
In euros^c^, median (IQR)	100 (50-200)	100 (50-200)	100 (50-200)
Missed days of work due to atopic dermatitis in the past 6 months			
Median (IQR)	3.0 (2-6)	3.5 (2-10)	3.0 (2-5)
Atopic dermatitis–related physician visits in the past 6 months			
Mean (SD)	2.7 (3.2)	2.6 (2.9)	2.9 (3.5)
Median (IQR)	2 (1-4)	2 (1-4)	2 (1-4)
eHEALS^d^			
Mean (SD)	29.62 (5.63)	29.85 (5.95)	29.38 (5.29)
Median (IQR)	29 (26-34)	29 (26-35)	29 (26-33)

a SCORAD: Scoring Atopic Dermatitis Index.

b Not answered.

c Conversion rate: €1=US $1.15.

d eHEALS: eHealth Literacy Scale.

## Discussion

This study was designed as a noninferiority trial comparing a digitally delivered care concept with routine care as available in well-served regions. Importantly, routine care did not mandate participation in structured face-to-face training. Current data may provide a distinct insight into real-world availability and uptake of patient education programs across centers and age groups, though this was not a prespecified study objective, and observations are restricted to the limited study period and study sites.

To decrease the burden of disease for patients with AD, patient training programs are essential. Independent of the format, patient training provides those affected with valuable information on self-management and coping. Increased understanding of the disease in general, as well as individual action plans to deal with exacerbations and psychosocial challenges, enables patients and caretakers to live with the disease confidently and safely. This benefit of patient training is acknowledged in several guidelines, including strong positive recommendations with regard to the use of verified multidisciplinary face-to-face psychoeducative programs such as from AGNES and ARNE [40]. The relevance of patient education programs is further reflected in the variety of studies evaluating different concepts and modes of care delivery [11-18]. During the recruitment phase of this study, we observed a high subjective burden of disease and need for care among all age groups, despite the fact that all study centers were located in urban areas with access to specialized care. This may reflect the need for easily accessible and individual training concepts as proposed in the framework of this study. However, before implementing new concepts of care, it is important to validate contents and test training formats for their efficacy. The project *ADCompanion* proposes a mixture of general disease-related contents mainly based on the curricula from AGNES and ARNE and individual counseling sessions to improve self-management, disease severity, and quality of life. The digital format enables patients to have remote access to these contents, independently from their area of residence. Easily accessible digital health interventions have also been evaluated successfully for other chronic diseases. For example, among patients with chronic kidney disease, those participating in a digital 12-week physical activity intervention reported significantly better quality of life than those in the waiting control group [41]. Patients with asthma who used a self-management app over 8 weeks had better asthma control (measured by the asthma control test) than a control group. However, attrition in this study was high [42]. Further, a recent systematic review showed that despite heterogeneous study designs, remotely delivered, nurse-led interventions are clinically effective for several chronic conditions including diabetes, cardiovascular diseases, cancer, mental health conditions, obesity, asthma, and musculoskeletal disorders [43]. Finally, instructive trainings for physical therapy have also been evaluated in a randomized controlled trial with patients experiencing chronic shoulder pain. Participants receiving in-person trainings and those receiving remote instructions both reported comparable improvements regarding function and symptoms underlining the viability of scalable remote trainings. Based on these encouraging results from the literature, this study will evaluate an interdisciplinary remote care concept for patients with AD to improve access to care, particularly in underserved regions, but also for patients and caregivers with limited access to in-person programs due to other reasons, for example, challenges in child care for single and/or working parents.

Naturally, the chosen study design has strengths and weaknesses. Strengths are the randomized controlled study design, as well as the fact that the multicenter setting covers different regions of Germany. Further, the study integrates seamlessly into the clinical care of AD patients, not affecting any of the medical treatments but offering an added value. Among limitations, we need to mention that due to the restriction to the German language, representability is not optimal. Further, the measure of disease severity (SCORAD) also contains 2 subjective visual analogue scales regarding itch severity and sleep impairment. This may blur the objective assessment of eczema severity by the study doctors, as these items are not objectively verifiable, which may add to the assumption that assessments could vary from physician to physician. Further, participants of the control group may have a stronger focus on their disease than they would have if not enrolled in the study due to the use of the diary and participation in a clinical study. This may lead to an unspecific clinical improvement. Finally, uptake of in-person patient training within the control group and observation period was limited, reflecting real-world constraints in availability and participation across centers. This makes a direct comparison between face-to-face training and remote counseling impossible. The choice of a noninferiority design must be interpreted in light of the pragmatic, real-world definition of routine care applied in this trial. As observed in this study, only a minority of participants in the control group attended an in-person training program, reflecting routine care rather than an idealized educational intervention.

Accordingly, the noninferiority margin of 4 points on the SCORAD scale was selected to compare the digital intervention against real-world routine care, not against guaranteed participation in structured face-to-face training. This margin is based on established thresholds for clinically relevant differences in SCORAD and remains appropriate for assessing whether a digitally delivered intervention can achieve comparable disease control under routine care conditions. Importantly, the noninferiority design also allows testing for superiority of the intervention compared to a control group.

Despite these limitations, this study has several important implications for future care models in AD. By integrating a validated educational curriculum with individualized digital counseling, ADCompanion reflects a scalable and patient-centered approach that may help close existing gaps in access to specialized training—particularly for families with limited time, mobility, or geographic proximity to expert centers. As health systems increasingly move toward hybrid and digitally supported models, the findings of this trial will provide robust evidence on how remote, interdisciplinary interventions can supplement established training programs and support the expansion of their reach. Ultimately, this study aims to inform the development of flexible care pathways that preserve the strengths of traditional face-to-face programs while leveraging digital solutions to enhance long-term disease management and patient empowerment.

## Supplementary material

10.2196/88025 Multimedia Appendix 1 Ethics approvals of all study centers.

10.2196/88025 Checklist 1 SPIRIT checklist.

## Abbreviations

AD: atopic dermatitis
AGNES: Arbeitsgemeinschaft Neurodermitisschulung e.V. (German Working Group on Atopic Dermatitis Training for Children)
ANCOVA: analysis of covariance
ARNE: Arbeitsgemeinschaft Neurodermitisschulung für Erwachsene e.V. (German Working Group on Atopic Dermatitis Training for Adults)
CONSORT: Consolidated Standards of Reporting Trials
COPEJU: Coping With Disease Questionnaire for Adolescents
COPEKI: Coping With Disease Questionnaire for Children
EASI: Eczema Area and Severity Index
eHEALS: eHealth Literacy Scale
GDPR: General Data Protection Regulation
JUCKJU: Itch Questionnaire for Adolescents
JUCKKI: Itch Questionnaire for Children
PO-SCORAD: Patient-Oriented Scoring Atopic Dermatitis Index
SCORAD: Scoring Atopic Dermatitis Index
SKINDEX-29: 29-Item Dermatology-Specific Quality of Life Questionnaire
SPIRIT: Standard Protocol Items: Recommendations for Interventional Trials
