# Supplementary material for: Remote Interdisciplinary Care for Atopic Dermatitis Using a Digital Care Concept (ADCompanion): Protocol for a Multicenter Randomized Trial
Source: JMIR Res Protoc. 2026 Aug 20;15:e88025. doi: 10.2196/88025 (PMC13492303; doi:10.2196/88025)
Supplement: Supplementary file 1 [file resprot-v15-e88025-s001.pdf]

CHARITÉ
UNIVERSITÄTSMEDIZIN BERLIN

Charité | Chariteplatz 1 | 10117 Berlin

Frau
Dr. med. Stephanie Dramburg
Klinik für Pädiatrie m.S. Pneumologie, Immunologie und Intensivmedizin
CVK

Ethikkommission
Ethikausschuss am Campus Virchow-Klinikum

Vorsitzende: PD Dr. E. Kaschina
Geschäftsführerin:
Dr. med. Katja Orzechowski

Postadresse
Campus Charité Mitte
Chariteplatz 1 | 10117 Berlin

T +49 30 450 517 222
F +49 30 450 7517 952
ethikkommission@charite.de

Berlin, 14.03.2023

ADCompanion – eine multizentrische, randomisierte, Nichtunterlegenheitsstudie zum Vergleich der Wirksamkeit eines digitalen Versorgungskonzepts für Patient:innen mit atopischer Dermatitis mit der Regelversorgung
Antragsnummer: EA2/009/23
Vorgang vom 03.03.2023, Eingang am 07.03.2023, per E-Mail am 28.02.2023

Sehr geehrte Frau Dr. Dramburg,

hiermit bestätigen wir den Eingang des Schreibens vom 03.03.2023 mit folgenden Anlagen:

- Ethikantrag, unterzeichnet am 28.02.2023
- Studieninformation und Einwilligungserklärung Eltern, Version vom 03.03.2023
- Studieninformation und Einwilligungserklärung Jugendliche, Version vom 03.03.2023
- Studieninformation und Einwilligungserklärung Erwachsene, Version vom 03.03.2023
- Studieninformation und Einwilligungserklärung Prozessevaluation Eltern, Version vom 03.03.2023
- Studieninformation und Einwilligungserklärung Prozessevaluation Jugendliche, Version vom 03.03.2023
- Studieninformation und Einwilligungserklärung Prozessevaluation Erwachsene, Version vom 03.03.2023
- Projektspezifische AGBs des App-Herstellers, Version vom 22.02.2023
- Fragebogen T0, Version vom 28.02.2023
- Fragebögen T0/T1 Prozessevaluation, Version vom 28.02.2023
- Flyer Rekrutierung Kinder und Jugendliche, Version vom 28.02.2023
- Flyer Rekrutierung Erwachsene, Version vom 28.02.2023

Die Auflagen laut Votum vom 06.02.2023 sind somit erfüllt.

Gegenüber den zeitgleich eingereichten Änderungen erhebt die Ethikkommission keine Einwände.
Wir wünschen viel Erfolg bei der Durchführung der o.g. Studie.

Mit freundlichen Grüßen

PD Dr. med. Kaschina
Vorsitzende

Gartenstraße 210 – 214
48147 Münster, Germany
Tel.: +49 (0)251 929 2460
Fax: +49 (0)251 929 2478
E-Mail: ethik-kommission@aekwl.de
www.ethik-kommission-wl.de

Ethik-Kommission Westfalen-Lippe · Gartenstraße 210 – 214 · 48147 Münster

Evangelisches Klinikum Bethel (EvKB)
Universitätsklinik für Kinder-und Jugendmedizin
Herrn Univ.-Prof. Dr. med. Eckhard Hamelmann
Burgsteig 13
33617 Bielefeld

nur per E-Mail:
Gabriele.Siekmann@evkb.de

20. April 2023 CS/jm

**Aktenzeichen (bitte immer mit angeben):**	**2023-230-b-S**
Titel des Forschungsvorhabens:	ADCompanion – multizentrische, randomisierte, Nichtunterlegenheitsstudie zum Vergleich der Wirksamkeit eines digitalen Versorgungskonzepts für Patient:innen mit atopischer Dermatitis mit der Regelversorgung
Hier:	02_Neuantrag als lokale EK, Antrag vom 13.04.2023

Sehr geehrter Herr Professor Hamelmann,

für das oben genannte Forschungsvorhaben haben Sie die Beratung durch die Ethik-Kommission beantragt und angegeben, dass für das Vorhaben bereits eine Beratung durch eine andere öffentlich-rechtliche, für die Beratung nach ärztlichem Berufsrecht zuständige Ethik-Kommission erfolgt ist. Die auswärtige Beurteilung liegt hier vor.

Die Ethik-Kommission erkennt diese Stellungnahme gemäß § 6 ihrer Satzung auch für den hiesigen Zuständigkeitsbereich an. Somit bestehen seitens der Ethik-Kommission

keine grundsätzlichen Bedenken ethischer oder rechtlicher Art

gegen die Durchführung des Forschungsvorhabens.

Die vorliegende Einschätzung gilt für das Forschungsvorhaben, wie es sich auf Grundlage der in Anhang 1 genannten Unterlagen darstellt, sowie für die in Anhang 2 aufgeführten Ärztinnen und Ärzte. Bitte geben Sie diesen das Beratungsergebnis zur Kenntnis.

Für die Entscheidung der Ethik-Kommission erhebt die Ärztekammer Westfalen-Lippe Gebühren nach Maßgabe ihrer Verwaltungsgebührenordnung. Über die Gebühren ergeht ein gesonderter Bescheid.

Vorsitzender: Univ.-Prof. Dr. med. W. E. Berdel
Stellvertretende Vorsitzende: Prof. Dr. med. Dr. phil. G. Brodner, Univ.-Prof. Dr. med. D. Föll, Prof. Dr. phil. C. Frantz

Allgemeine Hinweise:

Mit der vorliegenden Stellungnahme berät die Ethik-Kommission die der Ärztekammer Westfalen-Lippe angehörenden Ärztinnen und Ärzte zu den mit dem Forschungsvorhaben verbundenen berufsethischen und berufsrechtlichen Fragen gemäß § 15 Absatz 1 Berufsordnung ÄKWL.

Die Einschätzung der Ethik-Kommission ist als ergebnisoffene Beratung für den Antragsteller nicht bindend. Unabhängig von der vorliegenden Stellungnahme verbleibt die medizinische, ethische und rechtliche Verantwortung für die Durchführung des Forschungsvorhabens bei dessen Leiter, Auftraggeber und bei allen an dem Vorhaben beteiligten Ärztinnen und Ärzten.

Die Ethik-Kommission der Ärztekammer Westfalen-Lippe und der Westfälischen Wilhelms-Universität Münster ist organisiert und arbeitet gemäß den gesetzlichen Bestimmungen und den GCP-Leitlinien der ICH.

Mit freundlichen Grüßen

Univ.-Prof. Dr. med. Wolfgang E. Berdel
Vorsitzender der Ethik-Kommission

Anhang 1

Folgende Unterlagen haben der Ethik-Kommission vorgelegen:
Bei mehreren Versionen eines Dokuments bezieht sich die Bewertung auf die neueste:

Eingang	Datierung	Anlage
12.04.2023	12.04.2023	Ethikantrag (Zweitvotum) zur ADCompanion-Studie
12.04.2023	12.04.2023	Ethikantrag_Erstvotum_Ethikkommission Charite_ADCompanion-Studie
12.04.2023	12.04.2023	Ethikantrag_Zweitvotum_Univ-Prof. Dr. med. Hamelmann_EvKB_ADCompanion-Studie
13.04.2023	13.04.2023	AD Companion_Evaluationskonzept_signed
13.04.2023	13.04.2023	ADCompanion Flyer Erwachsene
13.04.2023	13.04.2023	ADCompanion Flyer Kinder
13.04.2023	13.04.2023	ADCompanion_ Studien-App
13.04.2023	13.04.2023	ADCompanion_Hanifin 2022 EASi User Guide
13.04.2023	13.04.2023	ADCompanion_InformedConsent_Eltern_121222
13.04.2023	13.04.2023	ADCompanion_InformedConsent_Erwachsene_121222
13.04.2023	13.04.2023	ADCompanion-InformedConsent_Kinder 7-12_121222
13.04.2023	13.04.2023	ADCompanion-Studienprotokoll Deutsch V1_19.12.22-signed
13.04.2023	13.04.2023	AW_ Ethikantrag ADCompanion-Studie_ unser Aktenzeichen 2023-230-b-S
13.04.2023	13.04.2023	Ethikantrag_Zweitvotum_Univ.-Prof. Dr. med. Hamelmann_EvKB_ADCompanion-Studie_oU
13.04.2023	13.04.2023	InformedConsent_Jugendliche 13-17_121222
19.04.2023	19.04.2023	ADCompanion_InformedConsent_Eltern_PAI_202304 (1)
19.04.2023	19.04.2023	ADCompanion_InformedConsent_Eltern_PAI_202304
19.04.2023	19.04.2023	ADCompanion_InformedConsent_Erwachsene
19.04.2023	19.04.2023	ADCompanion_InformedConsent_Jugendliche_PAI_202304
19.04.2023	19.04.2023	ADCompanion_InformedConsent_Prozessevaluation_Eltern_PAI_202304
19.04.2023	19.04.2023	ADCompanion_InformedConsent_prozessevaluation_Erwachsene_PAI_202304
19.04.2023	19.04.2023	ADCompanion_InformedConsent_Prozessevaluation_Jugendliche PAI_202304
19.04.2023	19.04.2023	ADCompanion-InformedConsent_Kinder 7-12_PAI_202304
19.04.2023	19.04.2023	AW_ Ethikantrag ADCompanion-Studie_ unser Aktenzeichen 2023-230-b-S

Anhang 2

Die Beratung gilt für die folgenden Ärztinnen und Ärzte im Zuständigkeitsbereich der Ethik-Kommission:

Prüfstelle: Evangelisches Klinikum Bethel (EvKB), Burgsteig 13, 33617 Bielefeld
Prüfer: Univ.-Prof. Dr. med. Eckhard Hamelmann
Prüferin: Dr. med. Eva Dippel
Prüferin: Dr. med. Kerstin Landwehr
Prüferin: Dr. med. Christina Schorlemer

Friedrich-Alexander-Universität
Medizinische Fakultät

Ethik-Kommission der FAU • Krankenhausstr. 12 • 91054 Erlangen

Universitätsklinikum Erlangen
Hautklinik
Stephanie Dierke
Ulmenweg 18
91054 Erlangen
Deutschland

Ethik-Kommission

Vorsitzender:
Prof. Dr. med. Renke Maas
Stellv. Vorsitzende:
Prof. Dr. med. Kerstin Amann

Geschäftsstelle:
Krankenhausstr. 12, 91054 Erlangen
Telefon: +49 9131 85-22270
+49 9131 85-26210
Fax: +49 9131 85-26021
E-Mail: ethikkommission@fau.de
Internet: www.ethikkommission.fau.de

Erlangen, 30.05.2023/ CB

Antrag №: **23-150-Bn** (bitte bei Schriftwechsel angeben)

Beratung nach § 15 Berufsordnung für Ärzte in Bayern

Projekt:	ADCompanion – eine multizentrische, randomisierte, Nichtunterlegenheitsstudie zum Vergleich der Wirksamkeit eines digitalen Versorgungskonzepts für Patient:innen mit atopischer Dermatitis mit der Regelversorgung.
Antragsteller:	Stephanie Dierke

Sehr geehrte Frau Dierke,

Ihre o.g. Antrag/Anfrage eingegangen am 06.04.2023, wurde auf Basis der vorgelegten Unterlagen (Anlage 1) geprüft.

Nach § 15 BayBOÄ bedarf es keiner nochmaligen Beratung, wenn das Forschungsvorhaben bereits von einer nach Landesrecht gebildeten Ethik-Kommission beraten wurde.

Sollten Sie aus anderen Gründen eine Stellungnahme der Ethik-Kommission der FAU Erlangen-Nürnberg benötigen bzw. falls Sie eine erneute Beratung des Antrags wünschen, so bitten wir um kurze Mitteilung.

Der Antragsteller hat die Kosten des Verfahrens zu tragen. Diese Kostenentscheidung beruht auf Art. 1 Abs. 1, Art 6, 8, 10 und 11 BayKG. Hierüber ergeht eine gesonderte Rechnung.

Mit freundlichen Grüßen

i.A. Dr. Cornelia Fahrenwaldt
Stellv. Leitung der Geschäftsstelle

Anlage 1:

1) Checkliste.pdf vom 06.04.2023
2) EA2_009_23 Votum vom 14.03.2023 ohne U.pdf vom 06.04.2023
3) EA02_009_23_Ethikantrag_Wiedervorlage.pdf vom 06.04.2023
4) ADCompanion-Studienprotokoll Deutsch 1V2_06.03.2023.pdf vom 06.04.2023
5) ADCompanion_InformedConsent_Erwachsene.pdf vom 06.04.2023
6) ADCompanion_InformedConsent_Prozessevaluation_Erwachsene.pdf vom 06.04.2023
7) ADCompanion_InformedConsent_Eltern.pdf vom 06.04.2023
8) ADCompanion_InformedConsent_Jugendliche.pdf vom 06.04.2023
9) ADCompanion_InformedConsent_Prozessevaluation_Jugendliche_17.02.2023.pdf vom 06.04.2023
10) ADCompanion_InformedConsent_Eltern.pdf vom 06.04.2023
11) ADCompanion_InformedConsent_Prozessevaluation_Eltern.pdf vom 06.04.2023
12) ADCompanion-InformedConsent_Kinder 7-12_121222.pdf vom 06.04.2023
13) Lebenslauf Prof. Sticherling.pdf vom 06.04.2023
14) Antragsformular Ethik.pdf vom 06.04.2023
15) ADCompanion-Studienprotokoll Deutsch 1V2_06.03.2023.pdf vom 06.04.2023
16) Antragsbestätigung.pdf vom 06.04.2023
17) Kurzbeschreibung.pdf vom 06.04.2023
18) EA02_009_23_Ethikantrag_Wiedervorlage.pdf vom 08.05.2023
19) Nia-ADCompanion-AGB_22.02.2023 (1).pdf vom 08.05.2023
20) EA2_009_23 Votum vom 14.03.2023 (1).pdf vom 08.05.2023
21) EA2_009_23 Votum vom 06.02.2023 (1).pdf vom 08.05.2023
22) Anschreiben Wiedervorlage EA2_009_23.pdf vom 08.05.2023
23) ADCompanion_InformedConsent_Prozessevaluation_Jugendliche_17.02.2023.pdf vom 24.05.2023
24) Nia-ADCompanion-AGB_22.02.2023.pdf vom 24.05.2023
25) Nia-ADCompanion-AGB_22.02.2023.pdf vom 24.05.2023
26) Nia-ADCompanion-AGB_22.02.2023.pdf vom 24.05.2023
27) ADCompanion-InformedConsent_Kinder_17.02.2023.pdf vom 24.05.2023
28) ADCompanion_InformedConsent_Prozessevaluation_Eltern_17.02.2023.pdf vom 24.05.2023
29) ADCompanion_InformedConsent_Eltern_17.02.2023.pdf vom 24.05.2023
30) Nia-ADCompanion-AGB_22.02.2023.pdf vom 24.05.2023
31) ADCompanion_InformedConsent_Jugendliche_17.02.2023.pdf vom 24.05.2023
32) ADCompanion_InformedConsent_Eltern_17.02.2023.pdf vom 24.05.2023
33) Nia-ADCompanion-AGB_22.02.2023.pdf vom 24.05.2023
34) ADCompanion_InformedConsent_Erwachsene_17.02.2023.pdf vom 24.05.2023
35) Nia-ADCompanion-AGB_22.02.2023.pdf vom 24.05.2023

TECHNISCHE UNIVERSITÄT DRESDEN

Ethikkommission

Ethikkommission an der TU Dresden
Fetscherstraße 74, 01307 Dresden

Frau
Katharina Blümlein
Klinik und Poliklinik für Dermatologie
Universitätsklinikum Carl Gustav Carus Dresden
Fetscherstraße 74
01307 Dresden

Prof. Dr. med.
Bertold Renner
Vorsitzender der Ethikkommission

Telefon: 0351 458-2992
Telefax: 0351 458-4369
E-Mail: ethikkommission@mailbox.tu-dresden.de

Dresden, 09.08.2023

Auflagenerfüllung

Studie:	ADCompanion – eine multizentrische, randomisierte, Nichtunterlegenheitsstudie zum Vergleich der Wirksamkeit eines digitalen Versorgungskonzepts für Patient:innen mit atopischer Dermatitis mit der Regelversorgung
unser AZ:	BO-EK-176042023 *(bitte stets angeben!)*
eingereicht von:	Katharina Blümlein *für Dr. med. Susanne Abraham - Klinik und Poliklinik für Dermatologie*

Sehr geehrte Frau Blümlein,

mit den ergänzenden Unterlagen zu o. g. Studie, die am 10.07.2023 hier eingegangen sind, wurden die Auflagen aus unserem Schreiben vom 23.06.2023 erfüllt. Im Ergebnis gehen wir davon aus, dass die App „Nia“ entsprechende der zugelassenen Zweckbestimmung angewendet wird.

Die darüber hinaus eingereichten Änderungen zu den Hinweisen aus o. g. Schreiben werden lediglich zur Kenntnis genommen. Diese wurden inhaltlich jedoch nicht geprüft, da hierfür keine Wiedervorlage erforderlich war.

Nach Auffassung der Ethikkommission an der Technischen Universität Dresden bestehen nunmehr gegen das Forschungsvorhaben

☒ keine Bedenken.

☐ keine Bedenken. Die im Einzelnen aufgeführten weiteren Hinweise bzw. Empfehlungen[1] sollten jedoch berücksichtigt werden. Eine erneute Vorlage der überarbeiteten Unterlagen ist nicht notwendig.

[1] *Hinweise und Empfehlungen sollen auf die moralische und juristische Verpflichtung hinweisen und in freier Entscheidung und Verantwortung zur Überprüfung und Anpassung der Studiendokumente anregen.*

Die allgemeinen Hinweise aus der mit Auflagen versehenen Erstberatung / Erstbewertung gelten entsprechend und sind zu berücksichtigen.

Die Ethikkommission an der TU Dresden stützt sich bei der Beurteilung der eingereichten Studienunterlagen insbesondere auf die Richtlinien der Deklaration des Weltärztebundes von Helsinki in der jeweils geltenden Fassung, auf die strahlenschutzrechtlichen Vorschriften und auf die allgemein anerkannten Richtlinien für „Good Clinical Practice" (GCP).

Die Arbeitsweise und die Zusammensetzung der Ethikkommission entspricht den jeweils geltenden gesetzlichen Regelungen bzw. Empfehlungen.

Es wird bestätigt, dass keine Mitglieder der Ethikkommission, die am o. g. Forschungsvorhaben beteiligt sind, am Beratungs- bzw. Bewertungsergebnis mitgewirkt haben oder in anderer Form beteiligt gewesen sind.

Wir wünschen Ihnen bei der Durchführung Ihres Forschungsvorhabens viel Erfolg.

Mit freundlichen Grüßen

im Auftrag

Constanze Muellender

Digital signiert von Constanze Muellender
DN: cn=Constanze Muellender, c=DE, o=Technische Universitaet Dresden, ou=Ethikkommission an der TU Dresden, email=constanze_theresa.muellender@tu-dresden.de
Datum: 2023.08.09 11:20:50 +02'00'

PD. Dr. med. A. Tausche-Wunderlich
Stellv. Vorsitzende der Ethikkommission

Ass. jur. Constanze Müllender
Stellv. Leiterin der Geschäftsstelle

MHH

Medizinische Hochschule Hannover
Ethikkommission
Vorsitzender: Prof. Dr. Bernhard Schmidt

Sekretariat:
Marion Lange
Telefon: 0511 532-3443

Liane Höft
Telefon: 0511 532-9812

Stefanie Kloos
Telefon: 0511 532-6061

Fax: 0511 532-16 3443
ethikkommission@mh-hannover.de

Carl-Neuberg-Straße 1
30625 Hannover
Telefon: 0511 532-0
www.mh-hannover.de

MHH Ethikkommission OE 9515
30623 Hannover

Frau
PD Dr. Annice Heratizadeh
Klinik für Dermatologie, Allergologie und Venerologie
OE 6600

Email: Heratizadeh.Annice@mh-hannover.de

03.04.2023/TKH

Nr. 10840_BO_K_2023
Ganzheitliche Versorgung von PatientInnen mit Atopischer Dermatitis - ADCompanion (DRKD00030902)

Beratungsergebnis: Ohne Bedenken

Sehr geehrte Frau Dr. Heratizadeh,

als Vorsitzender habe ich im Auftrag der Mitglieder der Ethikkommission Ihr Schreiben vom 27.03.2023 und die Angaben zu Ihrem Vorhaben geprüft.

Die Ethikkommission hat keine Bedenken gegenüber der Durchführung des Studienvorhabens. Wir schließen uns dem Vor-Votum an.

Der Antragsteller ist seiner berufsrechtlichen Pflicht entsprechend § 15 (1) Berufsordnung für Ärzte in Niedersachsen bzw. Dienstanweisung des Präsidiums der Medizinischen Hochschule Hannover (September 2012) nachgekommen. Eine Wiedervorlage wird erst bei Änderungen des Vorhabens nötig. Das Beratungsverfahren ist hiermit abgeschlossen.

Die Ethikkommission weist darauf hin, dass die ärztliche und juristische Verantwortung bei den jeweiligen Prüfärzten verbleibt.

Datenschutzrechtliche Aspekte von Forschungsvorhaben werden durch die Ethikkommission grundsätzlich nur kursorisch geprüft. Dieses Votum / diese Bewertung ersetzt mithin nicht die Konsultation des zuständigen Datenschutzbeauftragten.

Mit freundlichen Grüßen

Prof. Dr. Bernhard Schmidt
Vorsitzender der Ethikkommission

Nachrichtlich:
Fischer.Sascha@mh-hannover.de
Werfel.Thomas@mh-hannover.de

Technische Universität München | Ethikkommission

Klinikum rechts der Isar Technische Universität MÜnchen
Klinik und Poliklinik ür Dermatologie und Allergologie
Frau PD Dr. Christina Schnopp
Biedersteiner Str. 29
80802 München
Deutschland

München, 27.04.2023

Unser Zeichen: **2023-228-S-KH** (bitte bei Schriftwechsel angeben)

Beratung nach § 15 Berufsordnung für Ärzte in Bayern

Studientitel:	ADCompanion – eine multizentrische, randomisierte, Nichtunterlegenheitsstudie zum Vergleich der Wirksamkeit eines digitalen Versorgungskonzepts für Patient:innen mit atopischer Dermatitis mit der Regelversorgung.
Antragssteller:	PD Dr. Christina Schnopp

Sehr geehrte Frau Dr. Schnopp,

die Ethikkommission hat Ihren Antrag vom 18.04.2023 auf der Basis der vorgelegten Unterlagen geprüft.

Mit dem o.g. Forschungsvorhaben hatte sich bereits die Ethikkommission der Charité Berlin als erstvotierende Ethikkommission befasst.

Die Ethikkommission der Technischen Universität München schließt sich diesem Votum an.

Die ethische und rechtliche Verantwortung für die Durchführung dieser Studie verbleibt bei Ihnen. Änderungen in Organisation und Ablauf sind der Ethikkommission zur erneuten Prüfung einzureichen.

Die Ethikkommission erwartet, dass jedes Forschungsvorhaben mit Versuchspersonen vor

Rekrutierungsbeginn in eine öffentlich zugängliche Datenbank eingetragen wird.

Datenschutzrechtliche Aspekte von Forschungsvorhaben werden durch die Ethikkommission grundsätzlich nur kursorisch geprüft. Dieses Votum / diese Bewertung ersetzt mithin nicht die Konsultation des zuständigen betrieblichen oder behördlichen Datenschutzbeauftragten.

Die Ethikkommission empfiehlt bei Kooperationen mit anderen Einrichtungen grundsätzlich eine vertragliche Regelung. Wenden Sie sich zur weiteren Beratung bitte an Ihre für Verträge zuständige Abteilung.

Die Leitung des Klinikums rechts der Isar erwartet bei Studien, die durch die öffentliche Hand oder Stiftungen gefördert werden, die Datensicherheit über das medizinische Datenintegrationszentrum MeDIZ sicherzustellen. Wenden Sie sich deshalb in diesen Fällen an das Institut für Medizinische Informatik, Statistik und Epidemiologie unter www.mediz.med.tum.de.

Bitte beachten Sie, dass dieses Votum nur für die im Zuständigkeitsbereich der Ethikkommission der TUM tätigen Ärzte und Wissenschaftler gültig ist.

Mit freundlichen Grüßen

Prof. Dr. Georg Schmidt
Vorsitzender der Ethikkommission
Technische Universität München

Vorgelegte Unterlagen:

1) EA2_009_23 Votum vom 06.02.2023.pdf vom 18.04.2023
2) EA2_009_23 Votum vom 14.03.2023.pdf vom 18.04.2023
3) ADCompanion-Studienprotokoll Deutsch V2_06.03.2023.pdf vom 18.04.2023
4) ADCompagnion_MUC_InformedConsent_Eltern.pdf vom 18.04.2023
5) ADCompagnion_MUC_InformedConsent_Jugendliche.pdf vom 18.04.2023
6) ADCompagnion_MUC_InformedConsent_Prozessevaluation_Jugendliche.pdf vom 18.04.2023
7) ADCompagnion_MUC_InformedConsent_ Prozessevaluation_Erwachsene.pdf vom 18.04.2023
8) ADCompagnion_MUC_InformedConsent_Erwachsene.pdf vom 18.04.2023
9) ADCompagnion_InformedConsent_Kinder.pdf vom 18.04.2023
10) ADCompagnion_MUC_ InformedConsent_Eltern.doc.pdf vom 18.04.2023
11) ADCompagnion_MUC_InformedConsent_Prozessevaluation_Eltern.pdf vom 18.04.2023
12) Fördermittel_Konsortialpartner.pdf vom 18.04.2023
13) Weiterleitungsvertrag.pdf vom 18.04.2023
14) 20230411DrftTH_PZvertrag_ADCompanion_Version1_CS.pdf vom 18.04.2023
15) Evaluationskonzept_ADCompanion_23062022_signed.pdf vom 18.04.2023
16) Stellungnahme des Klinikdirektors.pdf vom 18.04.2023
17) CurriculumVITAEengl_clinical_studies.pdf vom 18.04.2023
18) Antragsformular für Sonstige Studien.pdf vom 18.04.2023
19) 2022-09-14 Förderbescheid.pdf vom 18.04.2023
20) Flyer_Seite_1.pdf vom 18.04.2023
21) Flyer_Seite_2.pdf vom 18.04.2023
22) nicht verfügbar - Versicherungsbestätigung.pdf vom 18.04.2023
23) nicht verfügbar - Versicherungsbedingungen.pdf vom 18.04.2023

Friedrich-Alexander-Universität
Medizinische Fakultät

Ethik-Kommission der FAU • Krankenhausstr. 12 • 91054 Erlangen

DIAKONEO KdöR
Cnopfsche Kinderklinik / Neonatologie, Pädiatrie
Frau Dr. Irena Neustädter
Sankt-Johannis-Mühlgasse 19
90419 Nürnberg
Deutschland

Ethik-Kommission

Vorsitzender:
Prof. Dr. med. Renke Maas
Stellv. Vorsitzende:
Prof. Dr. med. Kerstin Amann

Geschäftsstelle:
Krankenhausstr. 12, 91054 Erlangen
Telefon: +49 9131 85-22270
+49 9131 85-26210
Fax: +49 9131 85-26021
E-Mail: ethikkommission@fau.de
Internet: www.ethikkommission.fau.de

Erlangen, 05.06.2023/KK

Antrag Nr: **23-187-Bn** (bitte bei Schriftwechsel angeben)

Beratung nach § 15 Berufsordnung für Ärzte in Bayern

Projekt:	ADCompanion – eine multizentrische, randomisierte, Nichtunterlegenheitsstudie zum Vergleich der Wirksamkeit eines digitalen Versorgungskonzepts für Patient:innen mit atopischer Dermatitis mit der Regelversorgung.
Antragsteller:	Dr. Irena Neustädter

Sehr geehrte Frau Dr. Neustädter,

Ihre o.g. Antrag/Anfrage eingegangen am 11.05.2023, wurde auf Basis der vorgelegten Unterlagen (Anlage 1) geprüft.

Nach § 15 BayBOÄ bedarf es keiner nochmaligen Beratung, wenn das Forschungsvorhaben bereits von einer nach Landesrecht gebildeten Ethik-Kommission beraten wurde.

Sollten Sie aus anderen Gründen eine Stellungnahme der Ethik-Kommission der FAU Erlangen-Nürnberg benötigen bzw. falls Sie eine erneute Beratung des Antrags wünschen, so bitten wir um kurze Mitteilung.

Der Antragsteller hat die Kosten des Verfahrens zu tragen. Diese Kostenentscheidung beruht auf Art. 1 Abs. 1, Art 6, 8, 10 und 11 BayKG. Hierüber ergeht eine gesonderte Rechnung.

Mit freundlichen Grüßen

i.A. Dr. Cornelia Fahrenwaldt
Stellv. Leitung der Geschäftsstelle

Anlage 1:
1) ADCompanion-InformedConsent_Kinder 7-12_09.05.2023.pdf vom 11.05.2023
2) ADCompanion-Studienprotokoll Deutsch V2_06.03.2023.pdf vom 10.05.2023
3) ADCompanion_InformedConsent_Eltern_09.05.2023.pdf vom 11.05.2023
4) ADCompanion_InformedConsent_Jugendliche 13-17_09.05.2023.pdf vom 11.05.2023
5) ADCompanion_InformedConsent_Prozessevaluation_Eltern_09.05.2023.pdf vom 11.05.2023
6) ADCompanion_InformedConsent_Prozessevaluation_Jugendliche_09.05.2023.pdf vom 11.05.2023
7) Anschreiben Ethikkommision Erlangen.pdf vom 11.05.2023
8) Antragsbestätigung.pdf vom 11.05.2023
9) EA2_009_23 Votum vom 06.02.2023.pdf vom 10.05.2023
10) EA2_009_23 Votum vom 14.03.2023.pdf vom 10.05.2023
11) Kurzbeschreibung.pdf vom 11.05.2023
